# Combined SGLT-2 Inhibitor and Mineralocorticoid Receptor Antagonist Therapy: Effects on the Renin–Angiotensin–Aldosterone System in Heart Failure Treatment

**DOI:** 10.3390/ijms27198522

**Published:** 2026-09-24

**Authors:** Luigi Petramala, Luca Caprioni Grasso, Giuseppe Calise, Francesco Circosta, Tommaso Claudio Letizia, Luca Marino, Claudio Letizia

**Affiliations:** 1 Department of Translational and Precision Medicine, University “Sapienza”, 00185 Rome, Italy; luigi.petramala@uniroma1.it; 2 Department of Medical and Cardiovascular Sciences, University “Sapienza”, 00185 Rome, Italy; luca.caprionigrasso@uniroma1.it (L.C.G.); g.calise@policlinicoumberto1.it (G.C.); francesco.circosta@uniroma1.it (F.C.); tommaso.letizia@uniroma1.it (T.C.L.); claudio.letizia@uniroma1.it (C.L.); 3 Department of Mechanical and Aerospace Engineering, University “Sapienza”, 00185 Rome, Italy

**Keywords:** sodium-glucose co-transporter-2 inhibitors, mineralocorticoid receptor antagonists, kidney disease, albuminuria

## Abstract

Sodium–glucose cotransporter 2 inhibitors (SGLT2is), originally developed as glucose-lowering agents for type 2 diabetes mellitus, have increasingly demonstrated clinical benefits in patients with chronic kidney disease and heart failure. We retrospectively evaluated 114 patients with heart failure admitted to the Emergency Medicine Unit of Policlinico Umberto I, Sapienza University of Rome, Italy, between March and December 2025. Patients were stratified into three groups: initiation of combined SGLT2i and mineralocorticoid receptor antagonist (MRA) therapy (Group A), initiation of SGLT2i alone (Group B), or ongoing SGLT2i therapy for >12 months (Group C). Hemodynamic parameters, serum and 24 h urinary electrolytes, and hormonal parameters were assessed at admission (T0), 7 days (T1), and 30 days (T2). Baseline anthropometric and laboratory parameters were comparable among groups, except for baseline 24 h urinary glucose excretion, significantly increased in Group C compared to Groups A–B. Hypertension was the most prevalent comorbidity (74%), followed by atrial fibrillation (49%), dyslipidemia (47%), and coronary artery disease (39%). Groups A and B showed activation of the renin–angiotensin–aldosterone system, while Group A exhibited increased 24 h urinary sodium and chloride excretion and lower urinary albumin excretion. These findings confirm that SGLT2i therapy, particularly when combined with MRAs, may favorably influence natriuresis, chloruresis, and urinary albumin excretion in patients with heart failure.

## 1. Introduction

Sodium–glucose cotransporter 2 inhibitors (SGLT2is) were originally introduced as glucose-lowering agents for patients with type 2 diabetes mellitus (T2DM). In recent years their clinical role has expanded well beyond glycemic control, both for HFrEF and HFpEF [1,2,3,4]. Evidence from large cardiovascular and renal trials has progressively shown that SGLT2is provide meaningful benefits in patients with chronic kidney disease (CKD) and heart failure (HF), regardless of diabetic status. Consequently, SGLT2is are now considered a key component of contemporary HF therapy [5,6].

The mechanisms underlying these benefits are complex and not yet fully elucidated. Although inhibition of glucose reabsorption in the proximal tubule remains their primary pharmacological action, SGLT2i also influence sodium handling, renal hemodynamics, plasma volume, myocardial metabolism, inflammation, and cardiorenal interactions. In particular, by reducing proximal sodium and glucose reabsorption, these agents increase distal sodium delivery and restore tubule-glomerular feedback [7,8]. This leads to a reduction in intraglomerular pressure and promotes an early natriuretic and osmotic diuretic effect, which may contribute to decongestion in patients with HF. At the same time, the early loss of sodium and water may induce a compensatory neurohormonal response. Activation of the renin–angiotensin–aldosterone system (RAAS) may occur shortly after treatment initiation, especially in patients with acute HF, in whom volume status and neurohormonal balance are often unstable [9,10]. Although this response is usually transient, its short-term behavior may have clinical and pathophysiological relevance.

Mineralocorticoid receptor antagonists (MRAs) are also a fundamental part of HF treatment [11,12,13]. By blocking aldosterone activity, they reduce sodium retention, potassium wasting, myocardial fibrosis, vascular inflammation, and adverse cardiac remodeling [14,15,16]. However, their use can be limited by concerns regarding renal function and hyperkalemia, particularly in older patients and in those with CKD. Therefore, combining SGLT2is with MRAs is of particular interest. These two classes may act through complementary mechanisms, especially with regard to sodium balance, congestion, renal hemodynamic, and modulation of aldosterone-mediated effects.

Despite the established prognostic benefits of both drug classes, less is known about the early hormonal, renal, electrolyte, and urinary changes that occur after the initiation of SGLT2i therapy, particularly when these agents are started together with MRAs. Short-term variations in renin, aldosterone, urinary sodium and chloride excretion, glycosuria, albuminuria, and proteinuria may provide useful information on the early biological response to treatment.

Therefore, this retrospective cohort study aimed to evaluate early hormonal, renal, electrolyte, and urinary changes in patients hospitalized for acute HF initiating SGLT2i therapy—either alone or in combination with MRA—compared with patients receiving chronic SGLT2i therapy.

## 2. Results

Demographic, anthropometric, and laboratory parameters of all patients at admission (T0) are shown in Table 1. A total of 114 patients were retrospectively included in the study (66 males and 48 females, mean age of 76.8 ± 10.1 years). We showed no differences between the three study groups regarding anthropometric and laboratory parameters. In the overall cohort, HF indicators such as LVEF and NT-proBNP showed mean values of 44.7 ± 11.5% and 3458 ± 377 pg/mL, respectively; moreover, parameters related to left ventricular remodeling, such as PWT and IVS, were 11.2 ± 2.4 mm and 11.8 ± 2.7 mm, respectively.

The prevalence of major comorbidities in the overall patients is shown in Figure 1. Essential hypertension (EH) was the most frequent comorbidity associated with HF (74%), followed by atrial fibrillation (AF, 49%), dyslipidemia (47%), and coronary artery disease (CAD, 39%). Lower prevalence rates were observed for type 2 diabetes mellitus (T2DM), chronic kidney disease (CKD), chronic obstructive pulmonary disease (COPD), obstructive sleep apnea syndrome (OSAS), and stroke (37%, 33%, 33%, 12%, and 5%, respectively). No statistically significant differences among the three study groups were observed in the prevalence of EH, T2DM, dyslipidemia, CKD, COPD, or AF. Conversely, in Group B the prevalence of CAD (34%) and OSAS (3%) was significantly lower compared with Group A (44% and 25%, *p* < 0.05; respectively) and Group C (44% and 22%, *p* < 0.05; respectively).

The distribution of major chronic pharmacological treatments in the study population is summarized in Figure 2. Beta blockers and diuretics were the most commonly used drug classes in the overall population (61% and 53%), followed by the combination of ACEi and ARBs (49%), anticoagulants (47%), antiplatelets (37%) and calcium-channel blockers (21%). Group C showed no significant increased use of ACE inhibitors/ARBs (57%) compared with Groups A and B (38% and 50%), and lower administration of antiplatelet therapy (11%) with respect to Groups A and B (38% and 44%, *p* < 0.05; respectively).

Table 2 shows the biochemical behaviors during follow-up (T0, T1, and T2), and no differences were observed regarding kidney function and electrolytes levels.

In Table 3 we have reported changes in renin, aldosterone, and cortisol levels during the three observation time points. Interestingly, Group A and B showed a significant increased behaviors of renin at T1 and T2 compared with T0 (Group A: 29.6 ± 18.7 pg/mL and 32.8 ± 23.5 pg/mL vs. 21.6 ± 20.3 pg/mL; Group B: 38.2 ± 29.2 pg/mL and 34.5 ± 27.5 pg/mL vs. 22.6 ± 23.0 pg/mL; *p* < 0.05; respectively). The same pattern was also observed in aldosterone levels (Group A: 147.9 ± 70.4 pg/mL and 150.7 ± 45.8 pg/mL vs. 99.7 ± 51.2 pg/mL; Group B: 154.7 ± 101.2 pg/mL and 157.8 ± 102.7 pg/mL vs. 103.8 ± 74.5 pg/mL; *p* < 0.05; respectively).

Table 4 shows the analysis of 24 h urinary excretion of electrolytes, albumin, protein and glucose during follow-up. We have found a significant increase in 24 h urinary sodium excretion at T1 compared with T0 in the overall cohort (155.9 ± 71.4 vs. 121.1 ± 70.7 mmol/24h, *p* < 0.05). Interestingly, in Group A, we have found higher 24 h urinary sodium excretion at T1 and T2 (162.4 ± 36.1 and 141.3 ± 24.0 mmol/24h, respectively) than that observed at T0 (107.8 ± 57.3 mmol/24h, *p* < 0.05). Similarly, Group A showed higher 24 h urinary chloride excretion at T1 and T2 compared with T0 (132.6 ± 60.3 and 130.4 ± 45.8 vs. 95.8 ± 54.7 mmol/24h, *p* < 0.05; respectively). Finally, regarding 24 h urinary glucose excretion, Group C showed higher 24 h urinary glucose excretion at T0 (153.8 ± 12.9 mmol/24h) with respect to Groups A and B (68.2 ± 14.5 and 73.1 ± 13.4 mmol/24h, *p* < 0.05; respectively). Interestingly, at T2 Groups A and B showed higher 24 h urinary glucose excretion (103.2 ± 10.8 and 124.7 ± 11.2 mmol/24h) with respect to T0 (68.2 ± 14.5 and 73.1 ± 13.4 mmol/24h, *p* < 0.05, respectively); furthermore, Group B showed higher excretion at T2 with respect to Group A (124.7 ± 11.2 vs. 103.2 ± 10.8 mmol/24h, *p* < 0.05).

Figure 3 shows the percentage changes from baseline (T0) to the end of follow-up (T2) in hormonal parameters, kidney function, and 24 h urinary excretion across Groups A, B, and C. Groups A and B showed greater increased plasma aldosterone (44 ± 24% and 52 ± 22%, respectively) and renin levels (39 ± 36% and 53 ± 32%, respectively) compared with Group C (5 ± 12% and 11 ± 13%, respectively; *p* < 0.05). Similarly, 24 h urinary sodium excretion increased more markedly in Groups A and B (36 ± 20% and 22 ± 14%, respectively) than in Group C (−1 ± 6%; *p* < 0.05). Group A also showed a greater increase in 24 h urinary chloride excretion (32 ± 12%) compared with Groups B and C (6 ± 4% and 2 ± 4%, respectively; *p* < 0.05). Regarding 24 h urinary glucose excretion, Group B showed the largest increase (70 ± 24%), which was significantly greater than that observed in Group C (8 ± 7%; *p* < 0.05), whereas Group A showed increase (50 ± 35%), not rising statistical significance. Conversely, 24 h urinary albumin excretion decreased more markedly in Group A (−18 ± 12%) than in Groups B and C (−5 ± 10% and −4 ± 9%, respectively; *p* < 0.05). No significant between-group differences were observed in 24 h urinary protein excretion (−15 ± 34%, −9 ± 26%, and −1 ± 24% in Groups A, B, and C, respectively) or in eGFR (5 ± 16%, 2 ± 10%, and 1 ± 6%, respectively).

## 3. Discussion

Originally introduced for the treatment of T2DM and subsequently for CKD, the SGLT2i have become a cornerstone of therapy for HF, representing a true pharmacological revolution. These drugs have demonstrated an unexpectedly complex and versatile pharmacodynamic profile; the conceptual evolution that has accompanied the clinical use of gliflozins, from simple “glycosuric” drugs to potent cardio-reno-metabolic agents, represents one of the most fascinating developments in contemporary medicine.

Recent evidence highlights how SGLT2is, through the induction of natriuresis, can transiently activate the renin–angiotensin–aldosterone system (RAAS) [7,9]. In particular, Ansary et al. integrating both murine and human models suggested that the diuretic effect of SGLT2is is primarily driven by natriuresis rather than pure osmotic diuresis [7]: this early sodium loss induces a transient reduction in effective circulating volume, leading to compensatory activation of the systemic RAAS. Such short-term activation of the RAAS may have a detrimental effect because the increased circulating aldosterone levels may have a negative effect on filtration and a lower sodium excretion.

Following a brief follow-up period, the results of the present study confirm that the initiation of SGLT2i therapy, alone or in association with MRA, is associated with immediate activation of the RAAS. Specifically, after 7 days from first administration of SGLT2i, both Groups (Groups A and B) showed an increase in renin and aldosterone levels, reflecting a compensatory response to the reduction in plasma volume induced by osmotic diuresis and natriuresis. This observation suggests that combination therapy may enhance the downstream functional blockade of the RAAS at the mineralocorticoid receptor level, potentially reducing the activation of tubule-glomerular feedback and attenuating compensatory neurohormonal stimulation. Although the literature shows that RAAS activation tends to decrease and normalize approximately 6 months after the initiation of SGLT2i treatment [9], the use of MRA may counter-regulate this activation, increasing the efficacy of the therapy in terms of both cardioprotection and nephroprotection, potentially ensuring better medium- and long-term outcomes.

Our study, despite being conducted in a short time period, confirmed the results on the reno-protective capacity of SGLT2is, highlighting the reduction in proteinuria and microalbuminuria, as previously demonstrated by large-scale studies such as the CREDENCE study [17], the DAPA-CKD study [18], and the EMPA-KIDNEY study [19]. The reno-protective effect was attributed to the modulation of tubule-glomerular feedback, which leads to a reduced intraglomerular filtration pressure through vasoconstriction of the afferent arteriole. Furthermore, we observed a greater reduction in proteinuria and especially in microalbuminuria in Group A compared to Group B, suggesting an active role of the use of MRAs, likely due to the counter-modulation of the RAAS rebound activation following the use of SGLT2 inhibitors.

The study also confirmed the glycosuric effect of SGLT2 inhibitor therapy; all patients newly treated with SGLT2i showed increased urinary excretion of glucose; interestingly, Group A treated with an association of SGLT2is and MRAs showed reduced glycosuric effect with respect to Group B. Rather than on the potential beneficial effects of SGLT inhibitors, this aspect could have interesting implications regarding the adverse effects of this class of drugs. Over the last few years, increasing attention has been directed toward the possible adverse consequences of the greater glycosuria induced by SGLT2i. In a large meta-analysis, Liu et al. reported a significantly higher incidence of genital infections in patients treated with SGLT2is, with an approximately threefold increased risk compared with control groups, suggesting a direct relationship between urinary glucose excretion and susceptibility to local infections [20]. More recently, Kittipibul et al. also described a higher occurrence of genitourinary tract infections among patients receiving this class of drugs, further supporting the role of persistent glycosuria as a potential contributor to these complications [21]. Although the clinical benefit of SGLT2 therapy confirms the efficacy of this treatment, especially in the elderly population, the development of urinary tract infections could represent a hypothetical resistance to the prescription of this therapy. Within this framework, our findings may offer an interesting perspective. The reduced glycosuric effect observed in patients treated with the combination of SGLT2is and MRAs could represent a mechanism capable of attenuating the adverse events traditionally linked to excessive urinary glucose excretion. Interestingly, in our opinion this reduced glycosuria does not appear to interfere with the diuretic and decongestive benefits that make SGLT2i a cornerstone of HF therapy. Our hypothesis, supported by this study, is that MRA-SGLT2 combination therapy could improve the safety profile of the treatment, maintaining its cardiovascular efficacy.

Another finding consistent with the previous literature [22,23] was the natriuretic effect of SGLT2i; all patients newly treated with SGLT2i demonstrated increased urinary sodium excretion, but a notable observation was the significantly greater increase in urinary sodium and chloride excretion of sodium and chloride in Group A already evident after a week after treatment initiation (T1). At the 1-month follow-up (T2), patients in Group A showed greater increases in urinary sodium and chloride excretion compared with Group B. This observation may be relevant in light of previous studies investigating the renal effects of mineralocorticoid receptor antagonists. Ferreira et al. reported that spironolactone therapy in acute HF was associated with higher spot urinary sodium excretion [24], supporting the idea that MRAs may contribute directly to sodium handling and decongestion. Similarly, the ATHENA-HF trial was developed on the premise that intensified spironolactone treatment could improve natriuresis in patients with persistent congestion and diuretic resistance [25]. Experimental data from Bauersachs et al. had already shown a significant increase in sodium excretion after spironolactone administration in congestive HF models [26]. More recently, attention has shifted not only toward sodium balance but also toward chloride homeostasis, which is increasingly recognized as an important determinant of neurohormonal activation and diuretic responsiveness in HF [27]; therefore, the enhanced chloruretic response observed in patients receiving combined SGLT2i and MRA therapy may have pathophysiological implications extending beyond simple electrolyte excretion, potentially contributing to improved diuretic efficiency and congestion control. Furthermore, urinary electrolyte excretion may vary over time. Indeed, in the group taking long-term SGLT2 inhibitors (Group C), excretion values of these electrolytes increased at baseline but varied over time. During short-term follow-up, however, patients in Groups A and B showed an initial increase in excretion. All these trends reflect the effects on urinary excretion of these electrolytes, both short- and long-term [28,29].

Finally, in agreement with existing evidence [30], in our cohort no patients newly treated with SGLT2i developed severe hyperkalemia (>5.5 mEq/L). At T2, only 7% of patients in Group A exhibited potassium levels >5 mEq/L, and none of the patients showed potassium over 5.5 mEq/L, suggesting that concomitant treatment with SGLT2is and MRAs may mitigate one of the most concerning adverse effects associated with MRA therapy [31].

Although our data are encouraging, several limitations deserve discussion. First, the retrospective design of the study prevents any definitive temporal or causal inference, allowing only hypothesis-generating conclusions; although the study was retrospective, no significant baseline differences were highlighted in the three groups studied, not revealing a significant recruitment bias. Second, this was a single-center study, and therefore the generalizability of our findings to broader and more heterogeneous populations remains limited. Third, the relatively small sample size may have reduced the statistical power of the analysis and limited the ability to detect additional clinically relevant differences between groups; moreover, an a priori sample sizing calculation was not carried out which weakens the interpretation of non-significant findings.

Because long-term renal function and plasma aldosterone and angiotensin II receptor blocker (RAAS) levels are well known after SGLT2i and MRA administration, our study focused the effects of these medications in the short term, assessing hyperacute changes in the plasma RAAS and urinary excretion. Indeed, although early changes in renal function, electrolytes, natriuresis, and parameters related to the renin–angiotensin–aldosterone system (RAAS) can provide useful insights into mechanisms of action, longer follow-up is needed to determine whether these effects translate into lasting clinical benefits.

Future studies should therefore aim to confirm these preliminary findings in larger, multi-center cohorts. A prospective study design with predefined follow-up time points at 6 months, 1 year, and 2 years would allow a more robust assessment of long-term renal and cardiovascular outcomes, as well as a better characterization of the safety and efficacy profile of combined SGLT2i and MRA therapy.

## 4. Materials and Methods

### 4.1. Study Design

This retrospective cohort study involved the recruitment of 149 patients (74 men and 75 women) admitted to the Emergency Department of Policlinico Umberto I, Sapienza University of Rome, Italy, and subsequently hospitalized in the Emergency Medicine Unit of the same hospital between 1 March 2025 and 31 December 2025. Inclusion criteria were age ≥18 years, hospitalization for decompensated chronic HF, and availability of clinical, laboratory, and echocardiographic data. Patients with pregnancy, trauma-related admission, terminal-stage malignancy or acute kidney disease were not considered eligible for enrolment. Among the admission diagnoses, all patients had a diagnosis of HF established in accordance with the most recent guidelines, based on clinical history, laboratory findings, and echocardiographic data, within a multidisciplinary framework involving collaboration with a cardiologist. Of the 149 patients, 35 were excluded from the study because they had been receiving home therapy with SGLT2is for less than 12 months. The remaining 114 patients (66 men and 48 women, with a mean age of 76.8 ± 10.1 years) were stratified into three groups:

- Group A: Patients in whom combination therapy with SGLT2si and mineralocorticoid receptor antagonists (MRAs) was started;
- Group B: Patients in whom SGLT2i therapy alone was started;
- Group C: Patients who had already been receiving SGLT2i therapy at home for more than 12 months.

Regarding pharmacological treatment, we used these medications (available at our hospital): loop diuretics: furosemide (dosage used: 20–40 mg/day); MRA: potassium kanreonate (dosage used: 50–100 mg/day); SGLT2 inhibitor: dapagliflozin (dosage used: 10 mg/day).

The study design is shown in Figure 4.

### 4.2. Data Extraction from Medical Records

For all enrolled patients, the following data were extracted from medical records at Emergency Department admission (T0):

- Demographic data: Age, sex, body weight, and body mass index (BMI);
- Medical history data: Comorbidities and home pharmacological therapy;
- Laboratory analysis: Hemoglobin (Hb), mean corpuscular volume (MCV), platelet count (PLT), white blood cell count (WBC), neutrophil count, lymphocyte count, serum creatinine, estimated glomerular filtration rate by CKD-EPI equation, blood urea nitrogen (BUN)/urea, procalcitonin (PCT), C-reactive protein (CRP), fibrinogen, N-terminal pro–B-type natriuretic peptide (NT-proBNP), D-dimer, international normalized ratio (INR), activated partial thromboplastin time (aPTT), aspartate aminotransferase (AST), alanine aminotransferase (ALT), ferritin, transferrin saturation (%TSAT), blood glucose, low-density lipoprotein cholesterol (LDL-C), high-density lipoprotein cholesterol (HDL-C), triglycerides, serum uric acid.
- Transthoracic echocardiography parameters: Pulmonary artery systolic pressure (PASP), left ventricular ejection fraction (LVEF), atrial enlargement, pericardial effusion, left ventricular hypertrophy (LVH), heart disease, inferior vena cava (IVC) diameter, pleural effusion, and B-lines on lung ultrasound.

In the same patients, the following parameters were extracted from medical records at admission (T0), and at 7 days (T1) and 30 days (T2) after admission:

- Hemodynamic parameters: Systolic blood pressure (SBP), diastolic blood pressure (DBP), mean arterial pressure (MAP), mean heart rate (HR), mean respiratory rate (RR), and body temperature (T);
- Serum analysis of major electrolytes: Sodium (Na), potassium (K), calcium (Ca), magnesium (Mg), phosphate (PO_4_), and chloride (Cl);
- Serum creatinine measurement with subsequent estimation of glomerular filtration rate (eGFR) according to the CKD-EPI equation;
- 24 h urine collection, with analysis of major urinary electrolytes (Na, K, Ca, Mg, PO_4_, Cl), urinary proteins (albuminuria and proteinuria), and urinary glucose;
- Radioimmunoassay measurements of renin (direct renin concentration, DRC), aldosterone (plasma aldosterone, PA), and cortisol.

All these dosages were performed during a low-calorie, low-sodium diet, at rest for at least 30 min, in patients without acute renal failure and with normal potassium levels.

### 4.3. Statistical Analysis

The data are expressed as mean value and standard deviation (SD) and the assessment of the differences between the numerical sets was evaluated either by the Mann–Whitney U test or by one-way analysis of variance (ANOVA) applying the Fisher least significant difference post hoc test for multiple evaluations. Comparisons were presented for each group at the 3 different sampling times (T0, T1, T2) and between groups—at the same sampling time—using repeated-measures analysis of variance (ANOVA), followed by post hoc pairwise comparisons when appropriate. Possible false positive effects have been taken into account by means of Holm–Bonferroni correction in the multiple evaluations. A time × group interaction test was adopted to assess if the longitudinal changes in the primary outcomes significantly differ between study groups over time. Chi^2^ statistics were used to assess differences between categorical variables. Percentage changes were calculated individually for each patient as [(T2 − T0)/T0] × 100 and subsequently averaged within each treatment group. A *p*-value less than 0.05 was considered statistically significant. Statistical analysis was performed using dedicated statistical software SPSS (Statistical Package for Social Sciences, software, version 24; SPSS Inc., Chicago, IL, USA).

The present study was conducted in accordance with the Declaration of Helsinki guidelines and was also approved by the local ethical committee. Study design was clearly written in a layperson language and provided to each study participant. Written informed consent was obtained from all subjects.

## 5. Conclusions

Our study, conducted on a population affected by HF, treated with SGLT2is alone or with the addition of MRAs, highlights that the simultaneous use of both molecules induces a better clinical profile, related to a probable counter-regulation of the excessive RAAS rebound activation, associated with the consequent increased renal excretion of sodium and chloride and reduced glucose excretion, but ensuring greater short-term nephroprotection, with reduced protein and albumin urinary excretion.

## Figures and Tables

**Figure 1:**
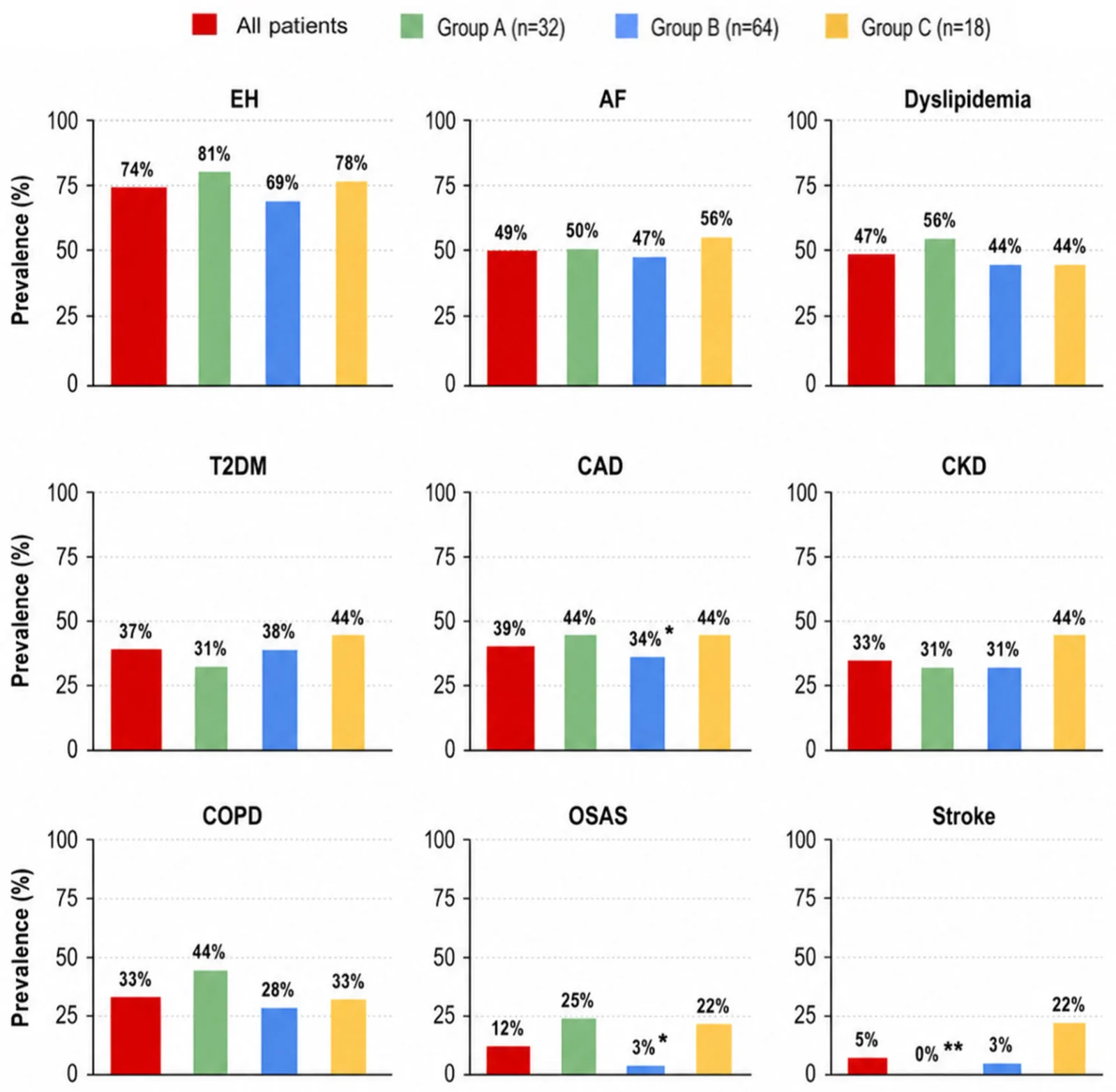
Prevalence of comorbidities among all patients enrolled. * = *p* < 0.05 vs. Group A and Group C; ** = *p* < 0.05 vs. Group B and Group C. EH: essential hypertension; AF: atrial fibrillation; T2DM: type 2 diabetes mellitus; CAD: coronary artery disease; CKD: chronic kidney disease; COPD: chronic obstructive pulmonary disease; OSAS: obstructive sleep apnea syndrome.

**Figure 2:**
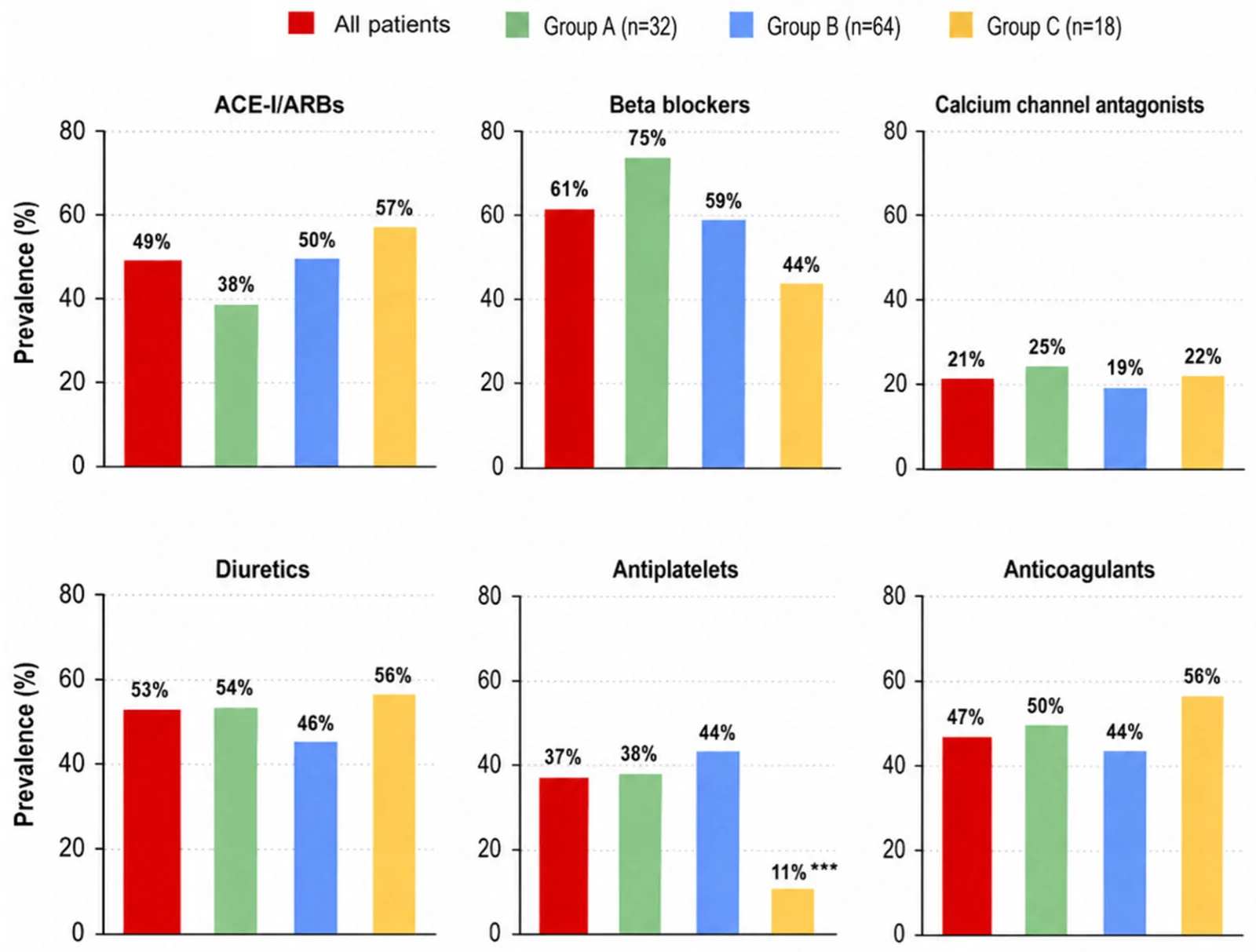
Prevalence of home pharmacological treatments among all patients enrolled. *** = *p* < 0.05 vs. Group A and Group B.

**Figure 3:**
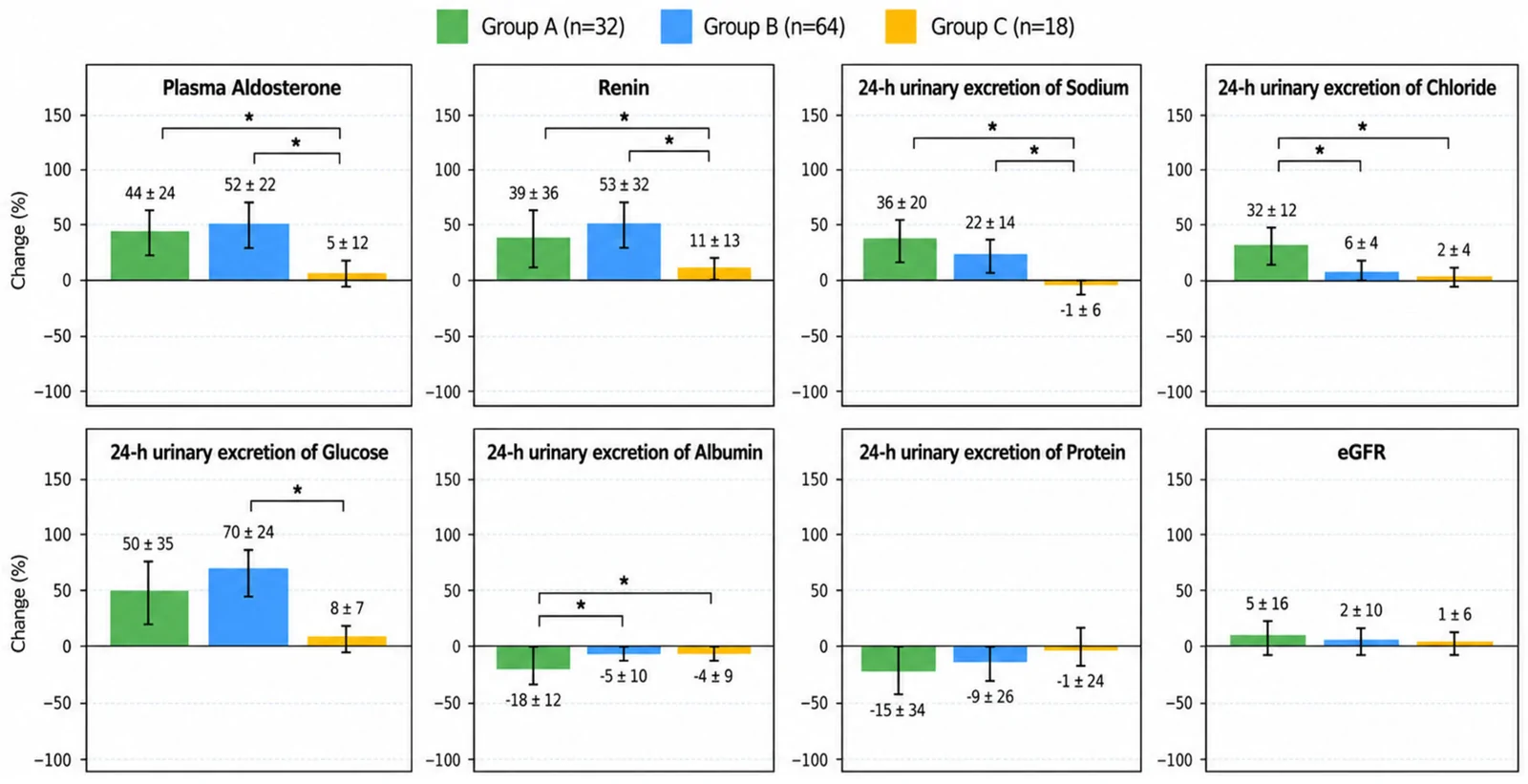
Changes (%) from baseline (T0) to the end of follow-up (T2) in hormonal parameters, kidney function, and 24 h urinary excretion of sodium, chloride, glucose, albumin, and protein. * = *p* < 0.05.

**Figure 4:**
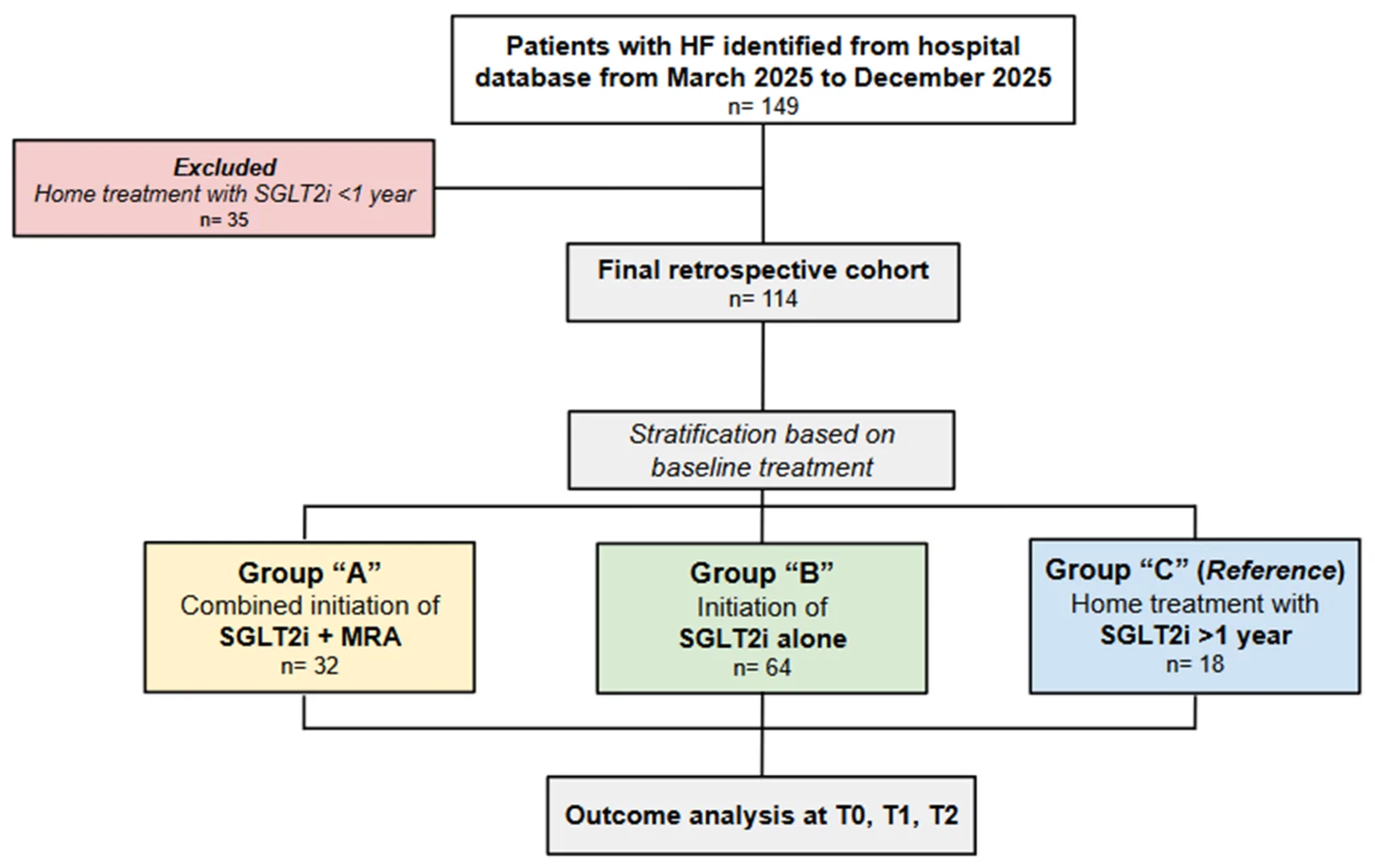
Retrospective cohort study design.

**Table 1:** Demographic, anthropometric, laboratory, and echocardiographic parameters of all patients enrolled at Emergency Department admission.

	All Patients (n = 114)	Group A (n = 32)	Group B (n = 64)	Group C (n = 18)	*p*-Value Between Groups A, B and C
**Age** (years)	76.8 ± 10.1	78.8 ± 8.0	75.8 ± 10.8	76.9 ± 10.0	n.s.
**Sex** (M/F)	66/48	20/12	36/28	10/8	n.s.
**BMI** (kg/m^2^)	25.9 ± 3.4	26.7 ± 3.7	25.4 ± 2.9	26.0 ± 4.3	n.s.
**SBP** (mmHg)	135.2 ± 20.0	131.3 ± 17.1	138.7 ± 22.0	129.4 ± 16.1	n.s.
**DBP** (mmHg)	75.7 ± 12.2	74.5 ± 11.4	76.8 ± 12.0	72.0 ± 13.5	n.s.
**HR** (bpm)	80.3 ± 17.5	80.1 ± 16.8	81.1 ± 18.8	77.8 ± 14.8	n.s.
**Creatinine** (mg/dL)	1.2 ± 0.4	1.3 ± 0.5	1.1 ± 0.3	1.4 ± 0.5	n.s.
**eGFR** (mL/min/1.73 m^2^)	63.4 ± 11.1	58.5 ± 9.6	67.4 ± 10.1	58.0 ± 15.9	n.s.
**Glucose** (mg/dL)	125.7 ± 9.8	124.3 ± 6.1	127.1 ± 12.8	123.4 ± 9.2	n.s.
**NT-proBNP** (pg/mL)	3458 ± 377	3257 ± 412	3673 ± 378	3050 ± 315	n.s.
**LVEF** (%)	44.7 ± 11.5	43.1 ± 11.1	46.7 ± 10.8	40.3 ± 14.5	n.s.
**Left atrial enlargement** (%)	63%	75%	56%	67%	n.s.
**PWT** (mm)	11.2 ± 2.4	10.9 ± 2.3	11.1 ± 2.5	12.3 ± 2.6	n.s.
**IVS** (mm)	11.8 ± 2.7	11.5 ± 2.6	11.7 ± 2.8	12.9 ± 3.0	n.s.

BMI = body mass index; SBP = systolic blood pressure; DBP = diastolic blood pressure; HR = heart rate; Creatinine = serum creatinine; eGFR = estimated glomerular filtration rate (CKD-EPI formula); Glucose = blood glucose; NT-proBNP = N-terminal pro-B-type natriuretic peptide; LVEF = left ventricular ejection fraction; PWT = posterior wall thickness; IVS = intraventricular septum thickness; Group A = patients initiated on combined SGLT2i and MRA therapy; Group B = patients initiated on combined SGLT2i alone; Group C = patients on stable SGLT2 inhibitor therapy for >1 year prior to enrollment; SGLT2i = sodium–glucose cotransporter 2 inhibitor; MRA = mineralocorticoid receptor antagonist; n.s.: not significant.

**Table 2:** Biochemical parameters of all patients enrolled at Emergency Department admission and during follow-up.

		All Patients (n = 114)	Group A (n = 32)	Group B (n = 64)	Group C (n = 18)	*p*-Value Between Groups A-B-C
**Creatinine** (mg/dL)	**T0**	1.2 ± 0.4	1.3 ± 0.5	1.1 ± 0.3	1.4 ± 0.5	n.s.
	**T1**	1.3 ± 0.4	1.4 ± 0.4	1.2 ± 0.4	1.3 ± 0.4	n.s.
	**T2**	1.2 ± 0.4	1.4 ± 0.5	1.2 ± 0.4	1.1 ± 0.5	n.s.
	*p-value between Groups T0, T1 and T2*	n.s.	n.s.	n.s.	n.s.	n.s. ^$^
**eGFR CKD-EPI** (mL/min/1.73 m^2^)	**T0**	63.4 ± 11.1	58.5 ± 9.6	67.4 ± 10.1	58.0 ± 15.9	n.s.
	**T1**	60.3 ± 22.3	55.6 ± 19.1	63.5 ± 22.9	57.6 ± 25.5	n.s.
	**T2**	64.2 ± 21.5	56.1 ± 19.0	66.5 ± 21.8	69.5 ± 22.8	n.s.
	*p-value between Groups T0, T1 and T2*	n.s.	n.s.	n.s.	n.s.	n.s. ^$^
**Sodium** (mEq/L)	**T0**	139.5 ± 3.7	139.9 ± 3.3	139.9 ± 3.5	137.2 ± 4.9	n.s.
	**T1**	139.5 ± 3.3	138.3 ± 3.0	140.3 ± 3.3	138.9 ± 3.4	n.s.
	**T2**	139.1 ± 3.2	138.1 ± 3.8	139.9 ± 2.9	137.4 ± 2.5	n.s.
	*p-value between Groups* *T0, T1 and T2*	n.s.	n.s.	n.s.	n.s.	n.s. ^$^
**Potassium** (mEq/L)	**T0**	4.0 ± 0.7	4.1 ± 0.4	3.9 ± 0.8	4.1 ± 0.9	n.s.
	**T1**	4.1 ± 0.7	4.3 ± 0.5	4.0 ± 0.8	4.1 ± 0.5	n.s.
	**T2**	4.0 ± 0.4	4.2 ± 0.4	4.0 ± 0.4	3.9 ± 0.6	n.s.
	*p-value between Groups* *T0, T1 and T2*	n.s.	n.s.	n.s.	n.s.	n.s. ^$^.
**Chloride** (mEq/L)	**T0**	99.1 ± 5.0	98.8 ± 4.0	98.9 ± 5.6	100.6 ± 4.6	n.s.
	**T1**	100.1 ± 3.9	98.8 ± 3.8	100.8 ± 4.0	99.4 ± 3.0	n.s.
	**T2**	102.3 ± 13.4	99.9 ± 4.8	101.3 ± 3.6	110.0 ± 32	n.s.
	*p-value between Groups* *T0, T1 and T2*	n.s.	n.s.	n.s.	n.s.	n.s. ^$^

eGFR CKD-EPI = estimated glomerular filtration rate calculated according to EPI-CKD formula (2009); Group A = patients initiated on combined SGLT2i and MRA therapy; Group B = patients initiated on combined SGLT2i alone; Group C = patients on stable SGLT2 inhibitor therapy for >1 year prior to enrollment; SGLT2i = sodium–glucose cotransporter 2 inhibitor; MRA = mineralocorticoid receptor antagonist. ^$^ = *p*-value of (time × group) interaction; n.s.: not significant.

**Table 3:** Hormonal behaviors of all patients enrolled at Emergency Department admission and during follow-up.

KERRYPNX		All Patients (n = 114)	Group A (n = 32)	Group B (n = 64)	Group C (n = 18)	*p*-Value Between Groups A, B and C
**Renin** (pg/mL)	**T0**	24.3 ± 23.7	21.6 ± 20.3	22.6 ± 23.0	28.9 ± 29.4	n.s.
	**T1**	34.9 ± 25.8	29.6 ± 18.7 *	38.2 ± 29.2 *	32.4 ± 56.1	n.s.
	**T2**	33.7 ± 25.1	32.8 ± 23.5 *	34.5 ± 27.5 *	32.1 ± 58.8	n.s.
	*p-value between Groups* *T0, T1 and T2*	n.s.	* *p* < 0.05 vs. T0	* *p* < 0.05 vs. T0	n.s.	
**Aldosterone** (pg/mL)	**T0**	103.3 ± 64.8	99.7 ± 51.2	103.8 ± 74.5	108.0 ± 49.8	n.s.
	**T1**	146.9 ± 55.6	147.9 ± 70.4 *	154.7 ± 101.2 *	117.3 ± 65.1	n.s.
	**T2**	148.9 ± 54.2	150.7 ± 45.8 *	157.8 ± 102.7 *	113.8 ± 59.0	n.s.
	*p-value between Groups* *T0, T1 and T2*	n.s.	* *p* < 0.05 vs. T0	* *p* < 0.05 vs. T0	n.s.	
**Cortisol** (µg/dL)	**T0**	10.4 ± 5.4	9.8 ± 6.2	11.4 ± 5.3	8.0 ± 2.5	n.s.
	**T1**	10.5 ± 5.3	8.9 ± 5.6	11.0 ± 4.4	11.4 ± 7.5	n.s.
	**T2**	9.6 ± 4.0	8.6 ± 3.7	9.9 ± 3.6	10.4 ± 5.8	n.s.
	*p-value between Groups* *T0, T1 and T2*	n.s.	n.s.	n.s.	n.s.	n.s. ^$^

Group A = patients initiated on combined SGLT2i and MRA therapy; Group B = patients initiated on combined SGLT2i alone; Group C = patients on stable SGLT2 inhibitor therapy for >1 year prior to enrollment; SGLT2i = sodium–glucose cotransporter 2 inhibitor; MRA = mineralocorticoid receptor antagonist. ^$^ = *p*-value of (time × group) interaction; n.s.: not significant.

**Table 4:** The 24 h urinary excretion of electrolytes, albumin, protein and glucose in all patients enrolled at Emergency Department admission and during follow-up.

		All Patients (n = 114)	Group A (n = 32)	Group B (n = 64)	Group C (n = 18)	*p*-Value Between Groups A, B and C
**Urinary Sodium** (mmol/24h)	**T0**	121.1 ± 70.7	107.8 ± 57.3	122.1 ± 73.3	141.4 ± 84.6	n.s.
	**T1**	155.9 ± 71.4 *	162.4 ± 36.1 *	153.5 ± 75.5	152.6 ± 101.4	n.s.
	**T2**	145.3 ± 55.8 *	141.3 ± 24.0 *	148.7 ± 58.5	140.1 ± 83.6	n.s.
	*p-value between Groups T0, T1 and T2*	* *p* < 0.05 vs. T0	* *p* < 0.05 vs. T0	n.s.	n.s.	***p*** **< 0.05 ^$^**
**Urinary Potassium** (mmol/24h)	**T0**	34.2 ± 19.8	41.3 ± 26.8	31.8 ± 17.8	30.2 ± 5.7	n.s.
	**T1**	39.6 ± 29.5	39.4 ± 30.8	41.7 ± 32.9	32.7 ± 5.9	n.s.
	**T2**	31.8 ± 13.3	32.0 ± 13.9	32.9 ± 14.0	27.3 ± 9.2	n.s.
	*p-value between Groups T0, T1 and T2*	n.s.	n.s.	n.s.	n.s.	n.s. ^$^
**Urinary Calcium** (mmol/24h)	**T0**	2.3 ± 1.7	1.6 ± 1.3	2.5 ± 2.1	2.7 ± 1.8	n.s.
	**T1**	2.5 ± 1.6	2.2 ± 1.8	2.5 ± 1.4	2.6 ± 1.8	n.s.
	**T2**	2.9 ± 5.5	4.8 ± 12.5	2.2 ± 1.9	2.1 ± 1.9	n.s.
	*p-value between Groups T0, T1 and T2*	n.s.	n.s.	n.s.	n.s.	n.s. ^$^
**Urinary Phosphorus** (mmol/24h)	**T0**	14.0 ± 8.8	16.4 ± 9.3	13.9 ± 9.5	10.6 ± 3.7	n.s.
	**T1**	16.3 ± 12.0	17.7 ± 17.4	17.3 ± 9.8	10.1 ± 4.8	n.s.
	**T2**	22.3 ± 39.7	38.6 ± 85.0	16.9 ± 8.4	14.6 ± 5.4	n.s.
	*p-value between Groups T0, T1 and T2*	n.s.	n.s.	n.s.	n.s.	n.s. ^$^
**Urinary Magnesium** (mmol/24h)	**T0**	2.5 ± 3.5	1.9 ± 1.2	2.9 ± 4.5	1.9 ± 1.2	n.s.
	**T1**	2.4 ± 1.4	2.7 ± 2.0	2.5 ± 1.1	1.8 ± 1.1	n.s.
	**T2**	2.9 ± 5.6	5.2 ± 19.6	2.3 ± 1.1	1.6 ± 0.7	n.s.
	*p-value between Groups T0, T1 and T2*	n.s.	n.s.	n.s.	n.s.	n.s.^$^
**Urinary Chloride** (mmol/24h)	**T0**	112.0 ± 62.5	95.8 ± 54.7	110.3 ± 62.7	146.8 ± 97.9	n.s.
	**T1**	132.5 ± 74.6	132.6 ± 60.3 *	127.7 ± 71.4	149.4 ± 81.9	n.s.
	**T2**	120.9 ± 54.7	130.4 ± 45.8 *	115.1 ± 55.9	125.6 ± 66.4	n.s.
	*p-value between Groups T0, T1 and T2*	n.s.	* *p* < 0.05 vs. T0	n.s.	n.s.	***p*** **< 0.05 ^$^**
**Urinary Albumin** (mg/24h)	**T0**	61.3 ± 85.0	100.1 ± 122.5	46.4 ± 64.9	45.5 ± 43.6	n.s.
	**T1**	64.8 ± 99.2	103.7 ± 107.8	51.9 ± 102.8	41.6 ± 43.4	n.s.
	**T2**	52.4 ± 82.6	81.8 ± 92.3	41.0 ± 85.4	43.7 ± 38.7	n.s.
	*p-value between Groups T0, T1 and T2*	n.s.	n.s.	n.s.	n.s.	n.s. ^$^
**Urinary Proteins** (mg/24h)	**T0**	239.7 ± 160.3	245.7 ± 167.0	232.4 ± 148.0	254.9 ± 195.6	n.s.
	**T1**	244.8 ± 192.1	251 ± 154.0	235.3 ± 213.2	267.6 ± 180.7	n.s.
	**T2**	217.2 ± 126.3	206.7 ± 111.6	211.3 ± 120.3	256.6 ± 166.6	n.s.
	*p-value between Groups T0, T1 and T2*	n.s.	n.s.	n.s.	n.s.	n.s. ^$^
**Urinary Glucose** (mmol/24h)	**T0**	84.5 ± 123.7	68.2 ± 139.8	73.1 ± 111.8	**153.8 ± 125.2 ^#^**	**^# ^*p* < 0.05 vs. A and B**
	**T1**	127.2 ± 127.1 *	95.9 ± 129.3	129.0 ± 121.6	176.4 ± 140.7	n.s.
	**T2**	119.7 ± 121.8	103.2 ± 60.2 *	124.7 ± 55.3 *^, #^	166.6 ± 138.6	**^# ^*p* < 0.05 vs. A**
	*p-value between Groups T0, T1 and T2*	* *p* < 0.05 vs. T0	* *p* < 0.05 vs. T0	* *p* < 0.05 vs. T0	n.s.	n.s. ^$^

Group A = patients initiated on combined SGLT2i and MRA therapy; Group B = patients initiated on combined SGLT2i alone; Group C = patients on stable SGLT2 inhibitor therapy for >1 year prior to enrollment; SGLT2i = sodium–glucose cotransporter 2 inhibitor; MRA = mineralocorticoid receptor antagonist. ^$^ = *p*-value of (time × group) interaction.

## Data Availability

The raw data supporting the conclusions of this article will be made available by the authors upon request.

## Abbreviations

The following abbreviations are used in this manuscript:

ACEi	Angiotensin-converting enzyme inhibitor
AF	Atrial fibrillation
ALT	Alanine aminotransferase
ANOVA	Analysis of variance
aPTT	Activated partial thromboplastin time
ARB	Angiotensin receptor blocker
AST	Aspartate aminotransferase
BMI	Body mass index
BUN	Blood urea nitrogen
CAD	Coronary artery disease
CKD	Chronic kidney disease
CKD-EPI	Chronic Kidney Disease Epidemiology Collaboration
COPD	Chronic obstructive pulmonary disease
CRP	C-reactive protein
DBP	Diastolic blood pressure
DRC	Direct renin concentration
eGFR	Estimated glomerular filtration rate
EH	Essential arterial hypertension
Hb	Hemoglobin
HDL-C	High-density lipoprotein cholesterol
HF	Heart failure
HR	Heart rate
INR	International normalized ratio
IVC	Inferior vena cava
LDL-C	Low-density lipoprotein cholesterol
LVEF	Left ventricular ejection fraction
LVH	Left ventricular hypertrophy
MAP	Mean arterial pressure
MCV	Mean corpuscular volume
MRA	Mineralocorticoid receptor antagonist
NT-proBNP	N-terminal pro-B-type natriuretic peptide
OSAS	Obstructive sleep apnea syndrome
PA	Plasma aldosterone
PASP	Pulmonary artery systolic pressure
PCT	Procalcitonin
PLT	Platelet count
PWT	Posterior wall thickness
RAAS	Renin–angiotensin–aldosterone system
RR	Respiratory rate
SBP	Systolic blood pressure
SD	Standard deviation
SGLT2i	Sodium–glucose cotransporter 2 inhibitor
SPSS	Statistical Package for the Social Sciences
T2DM	Type 2 diabetes mellitus
TSAT	Transferrin saturation
WBC	White blood cell count
